# Comparisons between different elements of reported burden and common mental disorder in caregivers of ethnically diverse people with dementia in Trinidad

**DOI:** 10.1371/journal.pone.0201165

**Published:** 2018-07-25

**Authors:** Nelleen Baboolal, Gershwin Davis, Robert Stewart, Jolie Ramesar, Amanda McRae

**Affiliations:** 1 The Department of Clinical Medical Sciences, Faculty of Medical Sciences, The University of the West Indies, St. Augustine Campus, Trinidad, W.I; 2 The Department of Paraclinical Sciences, Faculty of Medical Sciences, The University of the West Indies, St. Augustine Campus, Trinidad, W.I; 3 Institute of Psychiatry, Psychology and Neuroscience, King’s College London, London, United Kingdom; 4 South London and Maudsley NHS Foundation Trust, London, United Kingdom; 5 Department Pediatrics Hematology/Oncology, University of Florida, Gainesville, Florida, United States of America; 6 The Department of Preclinical Sciences, Faculty of Medical Sciences, The University of the West Indies, St. Augustine Campus, Trinidad, W.I; University of West London, UNITED KINGDOM

## Abstract

**Objective:**

Culture plays a significant role in determining family responsibilities and possibly influences the caregiver burden associated with providing care for a relative with dementia. This study was carried out to determine the elements of caregiver burden in Trinidadians regarding which interventions will provide the most benefit.

**Methods:**

Seventy-five caregivers of patients diagnosed with dementia participated in this investigation. Demographic data were recorded for each caregiver and patient. Caregiver burden was assessed using the Zarit Burden Interview (ZBI), and the General Health Questionnaire (GHQ) was used as a measure of psychiatric morbidity. Statistical analyses were performed using Stata and SPSS software. Associations between individual ZBI items and GHQ-28 scores in caregivers were analyzed in logistic regression models; the above-median GHQ-28 scores were used a binary dependent variable, and individual ZBI item scores were entered as 5-point ordinal independent variables.

**Results:**

The caregiver sample was composed of 61 females and 14 males. Caregiver burden was significantly associated with the participant being male; there was heterogeneity by ethnic group, and a higher burden on female caregivers was detected at borderline levels of significance. Upon examining the associations between different ZBI items and the above-median GHQ-28 scores in caregivers, the strongest associations were found with domains reflecting the caregiver’s health having suffered, the caregiver not having sufficient time for him/herself, the caregiver’s social life suffering, and the caregiver admitting to feeling stressed due to caregiving and meeting other responsibilities.

**Conclusions:**

In this sample, with a majority of female caregivers, the factors of the person with dementia being male and belonging to a minority ethnic group were associated with a greater degree of caregiver burden. The information obtained through the association of individual ZBI items and above-median GHQ-28 scores is a helpful guide for profiling Trinidadian caregiver burden.

## Introduction

In approximately fifty years, the world will be populated by over 2 billion people 60 years or older [1]. While living longer may be considered a significant medical achievement, the major consequence is that the risk for dementia increases with age [2,3]. Accordingly, by the middle of the century, it is expected that the number of people with dementia (PwD) will increase from today’s 46 million to 131 million [2,3]. While the majority receive home care by informal caregivers, this vital human resource could be on the decline [1–3]. First, worldwide, people are living longer and fertility rates are declining, which will cause a reduction in the caregiver pools [1–3]. Second, their dedication is not without consequence, as the level of stress and the burden associated with dementia caregiving is detrimental to their own health and emotional stability [4,5].

Customarily, the level of caregiver burden is determined using the Zarit Burden Interview (ZBI) [5]. Dementia caregiver-reported burden is associated with increased levels of depression and anxiety as well as increased use of psychoactive medications, poorer self-reported physical health, compromised immune function, and increased mortality [6–12]. For a number of years, much of what was known about caregiver burden was based on information obtained mainly from white North Americans [13,14]. While this provided fundamental information about the level of burden in dementia caregivers, it did not reflect essential cultural differences about caregiver experiences [15]. The percentage of elderly individuals with Alzheimer’s disease and other dementias in ethnic minority groups compared to North American whites is increasing. It is thus important to understand the cultural differences in dementia caregiving and its associated burden [16]. Dementia related behaviors are more prevalent in PwD from ethnic minorities [17], making the role of caregivers even more challenging and dangerous. These individuals prefer not to use support services such as nursing homes [17]. Informal caregivers are exceptional resources for dementia care. They contribute by ensuring quality home care, reducing the time that persons with dementia PwD are institutionalized and providing an annual estimated 225 billion dollars of unpaid care [18].

Trinidad and Tobago is a high income country with a gross national income per capita of (US$)16,2040 in 2016 and a multi ethnic population of 35.4 percent East Indian, 34.2 percent African and the remainder being of European, Chinese and mixed descent [19]. The country is also multi religious with denominations including Roman Catholic, Hindu, Anglican and Presbyterian. It is an aging nation, with more than 12 percent of the population aged 60 years and over [19] and situated in a region of the world where the older population is expected to grow the fastest, with a projected 71% increase in the population aged 60 years or over by 2050 [19]. High levels of cardiovascular risk factors are recognized at a national level, and this is especially important since the chronic stress of caregiving may contribute to elevated biomarkers of cardiovascular risk and impaired kidney function [20,21]. It is anticipated that the predicted longevity will be accompanied by increasing numbers of cases of dementia in this nation, which already has a high prevalence of dementia [22]. It is noted that, in persons 70–89 years old in Trinidad, the prevalence of dementia exceeds the same age ranges in North America and Latin America. The lack of established residential long term and assisted living facilities and inadequate primary care and caregiving programs that target persons living with dementia results in seniors in Trinidad and Tobago having to rely primarily on family support. It is thus essential to understand how caregivers in Trinidad and Tobago are coping with the caregiver burden [19].

Numerous interventions to reduce the caregiver burden have been proposed [7,23–26]; however, cultural variations associated with the dementia caregiving experience need to be considered [14,15,27]. In particular, it is essential to establish the cultural elements of caregiving that are causing the burden. These considerations will allow health care professionals and policy makers to better meet the needs of the caregivers they serve [14,15,23,28,29]. We have previously reported preliminary findings on a group of caregivers in Trinidad [30]. In this report, we present an in-depth analysis quantifying the degree of burden reported by dementia caregivers, evaluating factors associated with a higher burden and investigating the association of different components of caregiver burden with common mental disorder symptoms in the caregiver.

## Methods

### Sample

We carried out a cross-sectional study of 75 patients diagnosed with dementia and their caregivers. Participants were recruited over a one year period through convenience sampling from a memory outpatient clinic and from Alzheimer’s Association support groups. A diagnosis of dementia was confirmed by clinical assessment according to Diagnostic and Statistical Manual of Mental Disorders, 4th Edition, Text Revision (DSM IV TR) clinical diagnostic criteria [31], including administration of the Mini-Mental State Examination (MMSE) [32] and MRI or CT neuroimaging. Informed consent was obtained from all caregivers for provision of their own information and informed consent by proxy was obtained for the investigation of patient characteristics. The study was approved by the Ethics Committee of the Faculty of Medical Sciences, The University of the West Indies, St. Augustine.

### Measurements

Demographic characteristics of patients were recorded including age, gender, ethnicity, and marital status. Duration of dementia was determined from medical records, and MMSE score at assessment was considered as a covariate. Caregiver characteristics ascertained were age, gender, relationship to patient, cohabiting status, marital status, level of education, and occupation status. The Zarit Burden Interview (ZBI) [5], used to establish the degree of burden was self -administered for the most part, and caregivers were assisted only if they wanted clarification on the questions. This interview consisted of a 22-item questionnaire with a five-item response set ranging from “never” to “nearly always” graded on a scale from 0 to 4, according to the presence and intensity of an affirmative response. Its questions referred to the caregiver/patient relationship and evaluated the caregiver's health condition, psychological well-being, finances, and social life. Although ZBI categories are often imposed to categorize levels of burden [5], mean values were used in this analysis. In addition, the 28-item General Health Questionnaire (GHQ-28) was also completed by all caregivers [33] and was categorized into a binary variable according to whether scores were below (<47) or above (47+) the sample median. This approach was carried out for pragmatic reasons, in order to define a group at risk of a mental disorder and to compare associations with different elements of caregiver burden. This followed a previously recommended approach for populations in which GHQ screening properties are not known [34].

### Statistical analyses

Stata and SPSS software were used. Having described the sample and caregiver characteristics, mean (SD) ZBI scores were compared between grouped characteristics. The median age was used a priori as a cut-off, as the sample size was too small for defining subgroups. Linear regression models were constructed in Stata and used for testing significance; nonordered exposures with 3 or more groups were entered as fixed covariates applying likelihood ratio tests, and ordered exposures were tested as ordinal covariates on one degree of freedom. Associations between individual ZBI items and common mental disorder symptoms in caregivers were analyzed in logistic regression models, with above-median GHQ-28 score as a binary dependent variable, and individual ZBI item scores entered as 5-point ordinal independent variables. Odds ratios were calculated with 95% confidence intervals and strengths of associations for ZBI items were ranked according to the Nagelkerke R^2^ statistic derived from the logistic regression model. This R^2^ statistic is an approximation of the proportion of variance explained–i.e., ranging from 0–1 and with higher values reflecting exposures which have a stronger potential explanatory power. For illustrative purposes, we categorized strong associations on the basis of an R^2^ >0.20 and weak associations on the basis of an R^2^ <0.10, with moderate associations in between these limits.

## Results

Patient and caregiver characteristics are summarized in the first columns of Tables 1 and 2. The sample of people with dementia had a mean (SD) age of 77.6 (8.3) years (range 59 to 94). The mean (SD) estimated duration of clinical dementia was 4.3 (3.5) years (range 0.08 to 14), and the mean (SD) MMSE score was 13.0 (8.7) ranging from 0 to 26. The caregivers had a mean (SD) age of 57.3 (15.2) years (range 27 to 86), and the mean (SD) ZBI score was 24.3 (14.5), ranging from 0 to 63. ZBI scores were significantly greater for those caring for male patients and were greater at marginal levels of significance for female compared to male caregivers; higher ZBI scores were also strongly associated with above-median GHQ-28 scores in caregivers, but otherwise there were no significant associations with patient or caregiver characteristics (Tables 1 and 2).

**Table 1:** Patient characteristics and their associations with caregiver Zarit Burden Inventory (ZBI) scores.

Patient characteristic		Number (%)	Mean (SD) ZBI score for caregiver	p-value
Age	<79	39 (52.7)	21.8 (16.2)	
	79+	35 (47.3)	23.9 (13.1)	0.53
Gender	Male	19 (25.3)	29.0 (14.5)	
	Female	56 (74.7)	20.6 (14.3)	0.031
Ethnicity	African	36 (48.0)	20.7 (15.2)	
	East Indian	13 (17.3)	18.2 (13.6)	0.070
	Other	26 (34.7)	27.8 (13.5)	
Marital status	Single	6 (8.2)	18.2 (12.5)	
	Married	30 (41.1)	21.2 (15.0)	0.54
	Divorced	5 (6.8)	28.2 (2.2)	
	Widowed	32 (43.8)	24.5 (15.8)	
Dementia Duration	<3 years	41 (55.4)	22.4 (15.8)	
	3+ years	33 (44.6)	23.7 (13.3)	0.70
MMSE score	>21	10 (21.7)	17.2 (12.3)	
	10–21	22 (47.8)	20.6 (18.3)	0.19
	<10	14 (30.4)	25.5 (12.0)	

**Table 2:** Caregiver characteristics and their associations with Zarit Burden Inventory (ZBI) scores.

Caregiver characteristic		Number (%)	Mean (SD) ZBI score for caregiver	p-value
Age	50 or below	27 (36.0)	23.7 (15.8)	
	>50	48 (64.0)	22.2 (14.2)	0.66
Sex	Female	61 (81.3)	24.2 (15.1)	
	Male	14 (18.7)	16.6 (11.5)	0.082
Relation	Spouse	18 (30.0)	22.8 (13.8)	
	Child	32 (53.3)	23.2 (14.8)	0.97
	Other	10 (16.7)	22.1 (15.8)	
Cohabiting	No	24 (32.0)	20.4 (14.9)	
	Yes	51 (68.0)	23.8 (14.6)	0.35
Marital status	Never	15 (0.20)	21.9 (15.9)	
	Married	41 (54.7)	22.1 (15.8)	0.81
	Separated	14 (18.7)	23.4 (12.1)	
	Widowed	5 (6.7)	28.6 (9.8)	
Education	Primary or less	22 (29.3)	23.2 (14.1)	
	Secondary	35 (46.7)	21.5 (14.5)	0.81
	Tertiary	18 (24.0)	24.6 (16.4)	
Occupation	Full-time	32 (43.2)	20.2 (15.3)	
	Part-time	3 (0.04)	17.7 (9.6)	
	Unemployed	3 (0.04)	29.0 (10.5)	0.52
	Housewife	10 (13.5)	26.4 (12.7)	
	Retired	26 (35.1)	25.0 (15.3)	
GHQ score	<47	37 (50.0)	18.9 (13.4)	
	47+	37 (50.0)	27.2 (14.7)	0.013

Associations between different ZBI items and above-median GHQ-28 scores in caregivers are described and ranked in Table 3. The strongest associations were found with domains reflecting the caregiver’s health having suffered, the caregiver not having sufficient time for themselves, their social life suffering, and admitting to feeling stressed between caregiving and meeting other responsibilities. Moderate associations were found with reported strain, loss of control of life, wishing for someone else to take on the caregiving role, anger, and a feeling of dependence. Associations were weakest (R2<0.05) with reported expectations of care, feeling unable to care for much longer, uncertainty, negative effects on other relationships, lack of money, feeling that they could do a better job, fear of the future, uncomfortable having friends over, and feeling that they should be doing more.

**Table 3:** Associations between different ZBI items and above-median GHQ-28 scores.

ZBI item	Association with above-median GHQ-28 score	
	Odds ratio (95% CI)	Nagelkerke R^2^
^**¶**^**Do you feel your health has suffered because of your involvement with your relative?**	4.06 (2.18–7.54)*	0.39
^**¶**^**Do you feel that because of the time you spend with your relative that you do have enough time for yourself?**	2.87 (1.77–4.65)*	0.33
^**¶**^**Do you feel your social life has suffered because you are caring for your relative?**	2.48 (1.62–3.79)*	0.31
^**¶**^**Do you feel stressed between caring for your relative and trying to meet other responsibilities for your family or work?**	2.19 (1.48–3.24)*	0.26
^β^Do you feel strained when you are around your relative?	2.14 (1.34–3.43)*	0.17
^β^Do feel like you have lost control of your life since your relative’s illness?	2.21 (1.28–3.80)*	0.15
^β^Do you wish that you could just leave the care of your relative to someone else?	1.96 (1.16–3.23)*	0.11
^β^Do you feel angry when you are around your relative?	1.78 (1.16–2.79)*	0.11
^β^Do you feel like your relative is dependent upon you?	1.52 (1.11–2.09)*	0.11
^γ^Overall, how burdened to your feel in caring for your relative?	1.46 (1.03–2.07)*	0.07
^γ^Do you feel your relative asks for more help than needed?	1.45 (1.00–2.10)	0.06
^γ^Do you feel that you do not have as much privacy as you would like because of your relative?	1.46 (0.94–2.27)	0.05
^γ^Do you feel embarrassed over your relative’s behavior?	1.45 (0.96–2.20)	0.05
^γ^Do you feel that your relative seems to expect you to take care of her/him as if you were the only one she/he depended on?	1.27 (0.93–1.72)	0.04
^γ^Do you feel that you will be unable to take care of your relative much longer?	1.30 (0.88–1.94)	0.03
^γ^Do you feel uncertain about what you should do about your relative?	1.27 (0.88–1.84)	0.03
^γ^Do you feel that your relative currently affects your relationship with other family members or friends in a negative way?	1.38 (0.81–2.36)	0.02
^γ^Do you feel that you do not have enough money to take care of your relative, in addition to the rest of your expenses?	1.23 (0.88–1.74)	0.02
^γ^Do you feel like you can do a better job in caring for your relative?	1.20 (0.85–1.68)	0.02
Are you afraid what the future holds for your relative?	1.09 (0.79–1.50)	0.01
^γ^Do you feel uncomfortable about having friends over because of your relative?	1.25 (0.78–1.99)	0.01
^γ^Do you feel like you should be doing more for your relative?	0.99 (0.71–1.38)	<0.01

*p<0.05

¶Strong associations (R^2^>0.20)

βModerate associations (R^2^0.10–0.20)

γWeak associations (R^2^<0.10)

## Discussion

It is widely accepted that providing care for a relative with dementia can be a potent source of chronic stress and can cause deleterious consequences for both the physical and emotional health of caregivers [7–12]. The need to assess the burden on the caregiver is important since there are many associations between stress and disease. The burden of care is even more important to assess in chronic conditions such as dementia, where individuals may require long term care for as many as ten years or longer. Care activities include dealing with personal tasks such as getting dressed, bathing, and dealing with incontinence.

The preponderance of females with dementia in this study is consistent with international studies [2]. The ethnic group distribution shows some difference to that expected at a national level, with fewer participants of East Indian descent (17.3%) than 2011 Census norms (35.4%) [19]. Whether this is specific to the services sampled or reflects differences in help-seeking would require further research. It is noted that traditional East Indian families are multigenerational, suffer in silence, and do not disclose their true feelings. Similar to caregiver burden studies conducted in Latin America, China and India [10,35–37], we found that over 80% of caregivers were women, and the majority were adult children and spouses. In our sample, caregiver burden was significantly associated with the person with dementia being male and belonging to a minority ethnic group. The minority ethnic group were individuals who were not of East Indian or African descent. This is an interesting finding since previous reports that focused on ethnic and racial diversity in caregiving mention in part that caregivers of African descent (57%) were more likely to experience a greater burden from caregiving than whites (33%) [38]. In our study, whites would be part of the minority ethnic group and would be experiencing greater caregiving burden than individuals of African descent. These findings suggest that the burden is not related to the ethnicity but rather to an individual in a society belonging to an ethnicity that is in the minority. An increased level of burden was also detected in female caregivers at borderline levels of significance. Other studies have also reported increased caregiver burden in female caregivers [39–41], and it is well recognized that spouse caregivers in particular may become overburdened and depressed [39–41] as well as having an increased risk of developing dementia themselves [39–42].

Greater caregiver burden scores using the ZBI were associated with increased caregiver GHQ scores, a finding that has been reported in previous studies [43] We specifically sought to evaluate this further in the dataset, investigating whether individual ZBI items were associated with common mental disorder symptoms to a similar extent, or whether there were some aspects of caregiver strain that were more strongly associated with mental health than others. We found that there was indeed a high level of variation in the contribution of individual ZBI items to the overall association with caregiver common mental disorder symptoms. There was heterogeneity with respect to the distribution of the items. The associations with reported health having suffered because of the caregiving role, or with feeling stressed, may reflect reverse causality (i.e., high GHQ scores rendering people more likely to report these). In our group of caregivers, 47% were either employed fulltime or part time. The association with having insufficient time for oneself may reflect not only the intensity and duration of the individual caregiving situations but also the fact that employed dementia caregivers have to make major changes in their work schedule because of their caregiving responsibilities; sixty-five percent go in late, leave early or take time off [38]. The strong association with social life having suffered is potentially important as it might be used for both screening and intervention in this community, both of which require further evaluation. It theoretically contradicts the lack of association with feeling uncomfortable having friends over and with reported negative effects on relationships with other family members; however, it is possible that some caregivers were reluctant to report these factors because of social expectations, resulting in lower item accuracy. Interestingly, concerns about privacy, embarrassment about behavior and financial worries were not significantly associated with increased GHQ score, although reported strain, loss of control, anger and dependency were associated at moderate strength.

The relatively specific associations of certain features of strain with higher common mental disorder symptoms need to be clarified in independent samples, as the analyses were exploratory; however, they suggest that particular attention needs to be given to caregivers reporting restrictions in their social activity as well as those admitting to feelings suggesting a common mental disorder, including irritability and anger as well as low mood and anxiety. Resources to encourage sharing of the caregiving role may need to be considered as well as other means to help caregivers feel less isolated.

This study is the first to characterize caregiver burden of Trinidadian dementia caregivers. Communicating these data to health care professionals and policy makers could assist in the design of interventions that will assist our dementia caregivers to better cope with this valuable, but frequently challenging role. One of the limitations of the study is that the type of dementia and the stage of the disease were not identified, and this may also have implications for caregiver burden. As individuals progress to the end stage of the disease, they would require more supervision. The sample was also not a population-based sample but a convenient sample, which would limit generalizations about the conclusions. A future study on ‘caregiver burden’ would also benefit from employing qualitative methodology. However, despite this, the strength of our contribution to this area of study is that our report lays the ground work for developing possible screening test for caregiver strain in our multiethnic population.
